# A stance data set on polarized conversations on Twitter about the efficacy of hydroxychloroquine as a treatment for COVID-19

**DOI:** 10.1016/j.dib.2020.106401

**Published:** 2020-10-15

**Authors:** Ece C. Mutlu, Toktam Oghaz, Jasser Jasser, Ege Tutunculer, Amirarsalan Rajabi, Aida Tayebi, Ozlem Ozmen, Ivan Garibay

**Affiliations:** a Department of Industrial Engineering, University of Central Florida, United States; b Department of Computer Science, University of Central Florida, United States

**Keywords:** Coronavirus, COVID-19, Hydroxychloroquine, Opinion mining, Polarity, Social media, Stance classification, Twitter

## Abstract

At the time of this study, the SARS-CoV-2 virus that caused the COVID-19 pandemic has spread significantly across the world. Considering the uncertainty about policies, health risks, financial difficulties, etc. the online media, especially the Twitter platform, is experiencing a high volume of activity related to this pandemic. Among the hot topics, the polarized debates about unconfirmed medicines for the treatment and prevention of the disease have attracted significant attention from online media users. In this work, we present a stance data set, COVID-CQ, of user-generated content on Twitter in the context of COVID-19. We investigated more than 14 thousand tweets and manually annotated the tweet initiators’ opinions regarding the use of “chloroquine” and “hydroxychloroquine” for the treatment or prevention of COVID-19. To the best of our knowledge, COVID-CQ is the first data set of Twitter users’ stances in the context of the COVID-19 pandemic, and the largest Twitter data set on users’ stances towards a claim, in any domain. We have made this data set available to the research community via the Mendeley Data repository. We expect this data set to be useful for many research purposes, including stance detection, evolution and dynamics of opinions regarding this outbreak, and changes in opinions in response to the exogenous shocks such as policy decisions and events.

## Specifications Table

**Table:** 

Subject	Social Science, Health Informatics, Computer Science
Specific subject area	Twitter stance detection, Sentiment analysis,
	Polarization among in audiences
Type of data	Text (CSV-formatted)
How data were acquired	Twitter API
Data format	Raw, Filtered
Parameters for data collection	Keyword query: Coronavirus; Corona; COVID-19; Covid19;
	Sars-cov-2; COVD; Pandemic (case insensitive)
Description of data collection	We collected Twitter data for April 2020 with the specified
	keyword queries. We considered only root tweets (excluding retweets).
Data source location	http://www.Twitter.com
Data accessibility	https://doi.org/10.17632/38nyzyt9bz.1
	We adhere to Twitter’s terms and conditions by not providing the tweet
	JSON, but sharing the stance labels with the tweet IDs, so that the tweets
	can be rehydrated from the Twitter API.

## Value of the Data

• Our data set contains stance labels for more than 14 thousand tweets. COVID-CQ contains the stances of Twitter users towards a COVID-19-related pandemic claim, besides being the largest human-annotated stance data set for social media. We expect this data set to be useful in the understanding of human behavior on online social media.
• We believe that this data set can be used for many research purposes, including stance detection or evolution and dynamics of opinions regarding this outbreak, and changes in opinions in response to the exogenous shocks such as policy decisions, medical research publications, and events.
• Current data sets generally consider the semantic structure of the shared content only; however, the COVID-CQ data set is created via a joint annotation of tweets’ text and the shared URLs when the tweets were not self-explanatory. Accordingly, our data set challenges the prediction models for stance detection tasks to incorporate further information besides the text of the tweets. This data set can help the research community to develop state-of-the-art models for stance classification.

## 1 Data Description

The annotated COVID-CQ data set includes 14,374 original tweets (tweets/mentions/replies), which were generated by 11,552 unique users on Twitter. We excluded the retweets from our data set annotation. Table 1 illustrates the size of our data set and the frequency of tweets for each class, in which the *”Favor”* class with containing 6841 tweets is the largest class, followed by *”Against”* class with 4685 tweets, and finally, the smaller class is *”Neutral”* with 2848 tweets. Due to Twitter private information policies, we only shared *tweetID*’s and the corresponding stance labels. Therefore, researchers should collect the tweets from Twitter API according to the information provided. The procedure of data collection is described in Github^2^.

**Table 1:** The frequency of the annotated tweets belonging to each stance class.

Stance	Number of Tweets
Neutral	2848
Against	4685
Favor	6841
**Total**	**14,374**

**Fig. 1:**
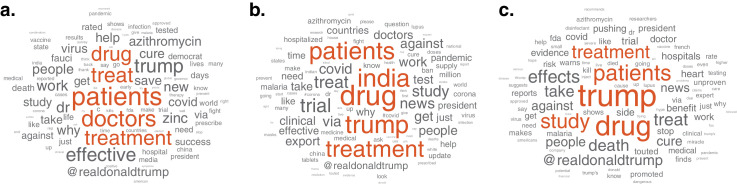
This figure demonstrates the most frequently used keywords in each stance category: (a) favor, (b) neutral, (c) against.

To briefly provide information on the underlying topics in our corpus, we demonstrated the most frequently used words for each stance category in Fig. 1.

These word clouds are achieved after preprocessing the textual content of the data set, including the elimination of stopwords and the common domain words, such as chloroquine, hydroxychloroquine, covid19, and coronavirus. A detailed explanation of text preprocessing and cleaning is provided in Section 4.3. Despite the high similarity of the content in our corpus, and the intertwined topics for all the three clusters (i.e. the topics related to the available drugs, hospitalized patients, and the treatment methods) (Table 2), it is clearly observable that some of the keywords have been appeared in a specific stance class in a higher frequency. Since a considerable number of tweets in the *Neutral/None* class are related to the import of the hydroxychloroquine drug from India to the US, the word “India” has appeared as one of the most frequent words in this class. For the *Favor* stance class, we observed that plenty of tweets in this category are related to the effectiveness of hydroxychloroquine as combination with two other drugs, Zinc and Azithromycin. Thus, the word cloud for the *Favor* class contains these keywords. Additionally, positive terms such as “effective”, “help”, “save”, and “success” are also more observable in the textual content of this category. Finally, the *Against* stance class has been observed to include words with a negative sentiment, including “risk”, “stop”, “warn”, “kill”, and “death”.

**Table 2:** The most common five words and their frequencies in each stance category.

Favor		Neutral		Against	
word	count	word	count	word	count
drug	1047	drug	467	Drug	1318
treatment	1099	treatment	326	Treatment	689
patient	1510	patient	354	Patient	906
doctor	1243	Trump	714	Trump	2863
treat	1180	India	673	Study	659

**Fig. 2:**
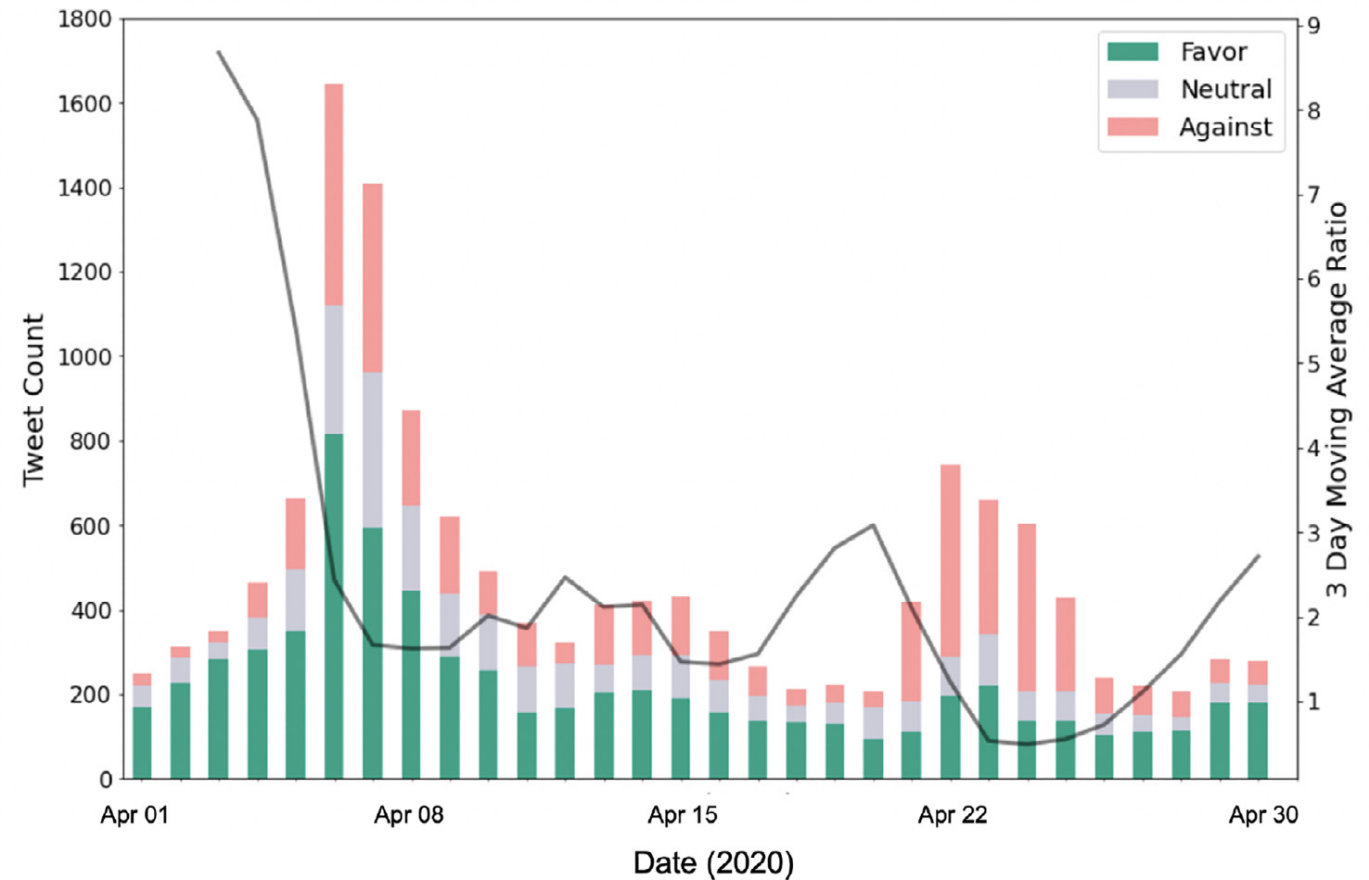
The daily tweet counts in April 2020, classified into three categories: ’neutral’, ’against’, and ’favor’. The black line refers to the ratio of the number of ’favor’ labeled tweets to the number of tweets with the ’against’ stance, for a 3 day moving average.

Figure 2 represents the daily counts of tweets that are labeled as *”Favor”* (green), *”Neutral”* (gray), and *”Against”* (pink) in a one month time period. As the month of April is the beginning of the chloroquine/hydroxychloroquine debate, the fluctuation in the ratio between *”Favor”* to *”Against”* tweets are found as very drastic; the black line demonstrates the 3-day moving average of the ratio of # of *”Favor”* tweets to # of *”Against”* tweets. Therefore, this data set offers not only a challenging corpus for the stance detection task, but also presents a drastic dynamic for the researchers who focus on a better understanding of the information diffusion, polarization, and opinion changes over time, in their studies.

Our proposed corpus differs from the reviewed data sets on various aspects. First, we only focus on the stances of Twitter users towards a single claim of “chloroquine and hydroxychloroquine are cure for the novel coronavirus”. Second, focusing on a single claim, our data set can be used to investigate the dynamics of opinions over time towards the use of these drugs, in response to the exogenous shocks such as academic publications and events. Thus, our data set fills the gaps between the changes of opinions over time and stance classification, which is not possible to investigate using existing stance data sets. Third, considering the challenges regarding the true inference of the underlying stance in a short text, we approached the problem of stance annotation as a joint labeling of tweet and shared URLs if the tweet is not self-explanatory. For instance, the Twitter platform imposes a maximum length of 280 characters for each tweet, which leads to the ambiguity of short tweets and the challenges for the inference of the true stances. As a result of this work, we aim to introduce new challenges to the field of artificial intelligence, particularly, to the design of stance data intensive classification models, and to encourage the design of algorithms that consider the utilization of all sources of information with the goal of achieving accurate stance classification of textual content. We believe that this data set can be used for many research purposes, including stance detection or evolution and dynamics of opinions regarding this outbreak, and changes in opinions in response to the exogenous shocks such as policy decisions, medical research publications, and events.

## 2 Major Events and Narratives

As the COVID-CQ data set is focused on a month-length Twitter activities regarding the COVID-19 pandemic ([7], and particularly, on the chloroquine/hydroxychloroquine conversations, this data contains textual content in relation to many major events that occurred since the beginning of the pandemic up to the end of April 2020. According to narrative summaries representing the ordered chain of individual events ([6]), below we briefly narrate the major events that appear in COVID-CQ data set. However, this narration does not reflect authors’ personal opinions towards any of the reviewed events.

i) The results of a study published on March 20 regarding the use of hydroxychloroquine to treat COVID-19 patients
  ([3]) have been widely discussed in our data set (referred to as “game changer” and “beginning of the end of the pandemic” by many Twitter users).
ii) The news on the purchase of hydroxychloroquine sulfate tablets by the Department of Veterans Affairs and the Bureau of Prisons in March 2020 are frequently discussed in our data set (examples of articles are New York Post^3^, The Daily Beast^4^, and Bloomberg^5^).
iii) The Emergency Use Authorization (EUA)^6^ for oral formulations of hydroxychloroquine sulfate and chloroquine phosphate was granted in March 28 by the Food and Drug Administration (FDA).
iv) The export ban of hydroxychloroquine by India was announced on March 25^7^. This decision was partially lifted on April 5 in response to a call from president Trump to Indian Prime Minister Narendra Modi^8^ (discussed in Twitter by using #retaliation)
v) The results of various clinical trials were announced during April 2020, from which, an international poll from 2,171 physicians started to propagate in Twitter conversations. This poll argues that 37% of doctors rated the drug hydroxychloroquine as the most effective therapy to combat the virus^9^.
vi) The immunity of patients with rheumatology illnesses against the novel coronavirus as a result of taking the drug hydroxychloroquine started to propagate on Twitter from April 7 (referring to Dr. Daniel Wallance’s report^10^ on 800 patients who take the drug).
vii) The relation of the white house with political and/or financial benefits from the use and support of hydroxychloroquine was widely discussed on Twitter^11^.
viii) The results published in a study regarding the patients at the US Veterans Health Administration medical centers^12^ suggested that patients taking hydroxychloroquine are no less likely to get infected by the virus, instead, the death rates in these peatiest have been observed to be higher ([5]).
ix) The FDA issued a warning^13^ about the use of this drug for COVID-19 patients on April 24 as a result of the the negative side effects of hydroxychloroquine that were reported in many research articles.

## 3 Experimental Design, Materials and Methods

### 3.1 Stance Annotation

Eisenberg and Finlayson define annotation as the ”process of explicitly encoding information about a text that would otherwise remain implicit” ([2]). For this study, annotation is the record of the Twitter audiences’ stances about the running debate of chloroquine and hydroxychloroquine as treatments for coronavirus. Our purpose is to create a pure human-annotated data set rather than ML-based data set with or without supervision, as we believe that human-annotation renders further studies more robust and reliable. The annotation procedure was conducted by a team of containing 6 graduate and 2 undergraduate students in order to reach a consensus on the annotation guideline. In our annotation procedure, each student was asked to annotate the individual tweets as *”Against”,”Favor” or ”Neutral/None”* for the unproven claim of *”chloroquine/hydroxychloroquine is cure for the novel coronavirus”*, relying on the well-known rumor listing website of fullfact.org^14^ as in Kwan et al.’s study ([4]). We have done our analysis on online users’ conversations regarding topics related to the COVID-19 pandemic in the Twitter platform. The data was collected using the Twitter API and Hydrator^15^ as suggested in ([1]). The detailed list of keywords being used for our data collection is given in the cited study. We considered tweets only related to the specific rumor and filtered tweets which include *”hydroxychloroquine, chloroquine, and HCQ”* as keywords for our queries, published between 04/01/2020 and 04/30/2020. This data set includes 98,671 tweets generated by 75,685 unique Twitter users. Since stances of the retweets may be easily attained with assumptions, we focused only on the 14,374 unique tweets (tweets/mentions/replies) generated by 11,552 unique users (The most active user has 91 user-generated contents.) to decrease the work-load.

**Fig. 3:**
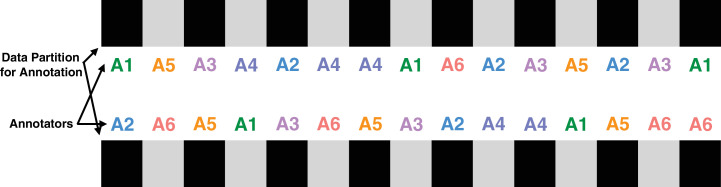
A visualization of the distribution of our data set for stance annotation.

Each of the investigated tweets is annotated by at least two different annotators. In the first round, the data was partitioned into 15 different clusters according to the time of the information creation and the tweet clusters were randomly assigned to the every possible combination of our annotator pairs. Thus, any possible biases due to annotator-pair match and time are prevented (Fig. 3). Among all the performed annotations, the inter-annotator agreement on this set was 87.37%, which demonstrates that our annotators were effectively educated before conducting the procedure. In the second round, the remaining 12.63% of the data was assigned to a third annotator who was not being asked to annotate that specific tweet for the first round. After this step, the majority vote was taken into consideration and label was determined with at least 2 out of 3 match. After labeling all tweets, the cosine similarity between td-idf tokenized and vectorized tweet texts and their labels were compared based on the assumption that similar tweets are more likely to have a similar stance. Then, all our annotators (8 people) were asked to discuss to reach a consensus on the inconsistent tweets. Thus, noisy labeling has been prevented.

Some of the challenges we faced while annotating this data are: i) Some of the tweets include irony/sarcasm; therefore, the true label might be hard to catch when the annotator is focused on the sentiment of the tweet only. ii) Since this debate is based on a health-related claim, the stances of a large amount of investigated tweets were ambiguous when the annotator only focused on the tweet text rather than a joint annotation of the tweet text and the shared URLs, if any. The source of this ambiguity is that many Twitter users shared URLs to the academic studies and news websites. iii) Each tweet can only contain up to 280 characters, which often poses a difficulty to fully understand the true meaning of the message, due to Twitter users being constrained to writing a short text. To overcome these challenges, we investigated the content of the URLs to understand the correct stances, only if the tweet text was not self-explanatory.

### 3.2 Annotation guidelines

We asked our annotators to label each of the tweets in our corpus using one of the three labels: *”Against”, ”Favor”, or ”Neutral/None”* regarding the unproven claim of *”Hydroxychloroquine and chloroquine are cures for the novel coronavirus.”*. To gain a deep understanding of the stances in tweets, and due to the high subjectivity related to this classification method, the ambiguous tweets were being discussed in details among the annotation team. In the following sub-sections, the three classification labels and their corresponding tweet examples are explained in more details.

I. **AGAINST:** This stance label was being used for the annotation of tweets that imply an opposition to the claim, either directly or indirectly.
  The stances of some of the tweets in this category are easily comprehensible as the tweet initiator expresses a direct opposition against the claim. For instance: *”I’m a physician. I would not take #Hydroxychloroquine for #COVID-19.”*.
  Some of the other tweets that were being identified as belonging to this category do not include personal opinions. Instead, these tweets might contain URLs to the academic studies or news articles in which hydroxychloroquine is demonstrated to be not effective against COVID-19, or simply contain the heading of the news article. It is assumed that the tweet initiator aims to share this information since he/she opposes the claim. An example of such a tweet is: *”No evidence of clinical efficacy of hydroxychloroquine in patients hospitalized for COVID-19 infection with oxygen requirement: results of a study using routinely collected data to emulate a target trial | medRxiv”*.
  Another example of expressing a counter attitude towards the aforementioned unproven claim, which was frequently observed in our data set, is via rationalizing against the claim. For instance, we observed that many Twitter users initiate contents that directly opposes the claim through expressing a reasoning, such as the side-effects of the drugs, or indirectly, via sharing the headings of news articles that imply the same concept:
  *”French Hospital Stops Hydroxychloroquine Treatment for COVID-19 Patients Over Major Cardiac Risk”*,
  *”Mr. Trump himself has a small personal financial interest in Sanofi, the French drugmaker that makes Plaquenil, the brand-name version of hydroxychloroquine.”*.
  Some tweets, on the other hand, include sarcasm/irony, which challenge the understanding of the true stance behind the textual content, even for human annotators. For instance: *”This is why you don’t take your medical advice from a reality TV host. #Hydroxychloroquine #COVID19”*.
II. **FAVOR:** This stance label was being used for the annotation of tweets that imply a support opinion towards the claim, either directly or indirectly.
  The stance label for some of the tweets in this category can be easily implied by the annotator, as the tweet initiator expresses a direct support in a straightforward language. For instance: *”It is TIME to open up businesses and schools again! Corona numbers are inflated and you know it! And...there’s a cure!...hydroxychloroquine!”*
  On the contrary to the above example, we observed that some of the tweet initiators convey their support of the claim in an indirect manner via questioning the conditions against what it seems to be an obvious solution to them. For instance: *Why did Fauci CHEER when hydroxychloroquine was used in 2013 for MERS, but is now skeptical for coronavirus?*
III. **NEUTRAL/NONE:** This stance label is the last category in the annotation of our data set, and its label was being used for the tweets that are neither in favor, nor against the aforementioned claim.
  We observed that most of the tweets in this category fall into one of these groups: i) tweets that are in the form of a question towards the truthiness of the claim, ii) tweets that convey a question with the aim of gaining more knowledge on the topic, iii) tweets that are written as a statement in a pure neutral tone, iv) tweets that only contain a neutral heading of a news article or an academic publication, and finally, v) tweets that contain the query keywords; however, no clear relation between the claim under study and the tweet content were implied.
  An example of the tweets which convey a question as in group ii discussed above, is: *”What is #Hydroxychloroquine ??? How does it work a against #Covid-19??”*
  Finally, the tweets that are focused on the events related to the chloroquine/hydroxychloroquine drugs, rather than a direct relation to the claim under study is: *”India sends hydroxychloroquine to UAE for COVID-19 patients.”*

### 3.3 Annotation Assessment

After the preparation of the data set according to the annotation guidelines, we conducted extensive analysis on the data to ensure the quality of the annotation, including the consistency of the stance labels in our corpus. In this regard, we investigated the semantic similarity of the tweets via computing the pairwise cosine similarity on the vector representation of the tweets. The Universal Sentence Encoder^16^ from the TensorFlow library was used to achieve the semantic vector representations of the tweets. The sentence level embeddings provide high level sentence semantic relationship, which enables the comparison of the similarity of tweet contents against each other to assure labeling consistency. After the calculation of pairwise cosine similarity for the tweets in our corpus, we reevaluated the labels of the tweets with  ≥ 0.9 cosine similarity, where this threshold was being identified by human judgement via comparing the tweet similarity results. The reevaluation procedure of the identified highly similar tweet contents include manual investigation of these tweets by the annotators, such that the highly similar tweets that convey the same stance towards the claim are categorized into the same stance class.

#### 3.3.1 Stance classification

To demonstrate the potential of evaluating many stance classification models using this data, and to evaluate the quality of our data set, we conducted extensive analysis using six different classification methods. The implemented models for this purpose include Multilayer Perceptron (MLP), Logistic Regression (LR), Support Vector Machine (SVM), Multinomial Naive Bayes (MNB), Stochastic Gradient Descent (SGD), Gradient Boosting (GB), and finally, Convolutional Neural Network (CNN). Furthermore, all the classification methods are compared for the computed vector representations of word unigrams and bigrams using the term frequency-inverse document frequency (tf-idf).

The implemented Multilayer Perceptron contained 2 dense layers, the rectified linear unit (ReLU) as the activation function, and the cross-entropy loss as the loss function. For the Logistic Regression classifier, the Limited-memory Broyden Fletcher Goldfarb Shanno (lbfgs) was used as the solver. The Support Vector Machine model was implemented with the linear kernel. For the Stochastic Gradient Descent model, the perceptron loss was used. In the implementation of the Gradient Boosting model, the deviance loss was used for model optimization. Finally, the Convolutional Neural Network (CNN) was implemented in two different ways to receive the inputs as vectors computed by one-hot encoding and GloVe word embedding, both with 5 convolutional layers with kernel size of 3 and stride size of 2, and with Exponential Linear Unit (ELU) activation function. The training stop criteria was to reach to a maximum number of 1000 iterations in training for all the classifiers.

#### 3.3.2 Data preprocessing

To prepare the input to the classifiers, we first filtered the tweets that were identified by Twitter to be in a different language than in English. After excluding the non-English tweets, we removed any punctuation marks and non-Ascii characters, and replaced the integers with their textual representation. Further preprocessing of the data include mapping all the input text to lowercase format, followed by word stemming, lemmatization, and the removal of the stopwords. Additionally, the URLs, hashtag signs (#*xxx*), and emoticons were removed to achieve a higher textual quality. It should be noted that none of these classification methods have been trained or tested on the content of the shared URLs as part of the input data. After this step, the term frequency-inverse document frequency (tf-idf) was used to generate the vector representation of the input tweets. Using tf-idf, we generated a vector space with weighting scheme based on the frequency of unigrams and bigrams in a tweet relative to the total number of their frequencies in the entire data set. Thus, tf-idf captures the most distinct words while ignoring the semantic or syntactic attributes. After this step, the vectorized tweets were used as the input to all the classifiers. For the training and testing of all the models, we used 80% of the tweets in training, and the remaining of the tweets to evaluate the models.

#### 3.3.3 Preliminary analyses

The comparison of the results for 6 classification models using our stance data set is provided in Table 3. Among the investigated classification methods, the Logistic Regression model achieved the best accuracy of 0.76 for both accuracies when the feature vectors for the tweets were computed using unigrams and bigrams for tf-idf. The next best performance was achieved by the SVM with very close performance to LR. The gradient boosting model achieved the lowest performance for both unigram and bigram tf-idf vectorized inputs. Surprisingly, using the bigrams to generate the tf-idf feature vectors did not affect the accuracy, observed for all models. However, despite the use of general purpose classification methods than state-of-the-art stance detection models, and although the contents from the URLs were not used for the evaluation of these classifiers, all of the models were able to classify the tweets with an acceptable performance. For further analysis, we implemented a Convolutional Neural Network (CNN) classifier for stance classification with one-hot encoding and GloVe vectorization of the words in tweets. This classifier achieved the accuracy of 0.73 for both vectorization methods. Additionally, the stance classification using the MLP model was also repeated for the one-hot encoded tweets. The achieved classification accuracy for this classifier was 0.75, which is slightly improved comparing with the MLP model using the tf-idf feature vectors.

**Table 3:** The comparison of the results for 6 classifiers using tf-idf vectorization.

Classifier	Unigram	Bigram
Stochastic Gradient Descent (SGD)	0.7429	0.7439
Support Vector Machine (SVM)	0.7651	0.7651
Multilayer Perceptron (MLP)	0.7453	0.7457
Logistic Regression (LR)	**0.7683**	**0.7683**
Multinomial Naive Bayes (MNB)	0.7182	0.7182
Gradient Boosting Classifier (GB)	0.6764	0.6768

## Ethics Statement

Our data does not provide any personally identifiable information and only the tweet IDs and human annotated stance labels are shared. Thus, our data set complies with Twitter’s information privacy policy.

## Declaration of Competing Interest

None.

## Footnotes

2 https://github.com/DocNow/hydrator

14 https://fullfact.org

15 https://github.com/DocNow/hydrator

16 http://www.tensorflow.org/hub/tutorials/semantic_similarity_with_tf_hub_universal_encoder

## Footnotes

3 http://www.nypost.com/2020/04/07/federal-agencies-purchase-large-supply-of-hydroxychloroquine/

4 http://www.thedailybeast.com/the-bureau-of-prisons-just-bought-a-ton-of-hydroxychloroquine-trumps-covid-19-miracle-drug?ref=scroll

5 http://www.bloomberg.com/news/articles/2020-03-20/hospitals-stockpile-malaria-drug-trump-says-could-treat-covid-19

6 http://www.fda.gov/media/136534/download

7 http://www.statnews.com/pharmalot/2020/03/25/india-trump-hydroxychloroquine-coronavirus-covid19/

8 http://www.bbc.com/news/world-asia-india-52196730

9 http://www.nypost.com/2020/04/02/hydroxychloroquine-most-effective-coronavirus-treatment-poll/

10 http://www.youtube.com/watch?v=kd7Jec3pZBk

11 http://www.washingtonpost.com/opinions/2020/04/07/real-reason-trump-is-obsessed-with-hydroxychloroquine/

12 http://www.cnn.com/2020/04/21/health/hydroxychloroquine-veterans-study/index.html

13 http://www.time.com/5827085/fda-warning-hydroxychloroquine/
